# C-951-06. Metabolomic Profiling of Lipid and Metabolic Alterations in Burn Injury: Identifying Biomarkers for Improved Management

**DOI:** 10.1093/jbcr/irag033.114

**Published:** 2026-04-06

**Authors:** William M Melito, Athena M Hoppe, Alison A Smith, M Victoria P Miles, Jonathan E Schoen, Jeffrey M Carter, Dhanushka Vitharana, Abdul-Razak Masoud, Jiri Adamec, Sabyasachi Chatterjee

**Affiliations:** University Medical Center Burn Center, Louisiana State University Health Sciences Center, New Orleans, LA; University Medical Center Burn Center, Louisiana State University Health Sciences Center, New Orleans, LA; University Medical Center Burn Center, Louisiana State University Health Sciences Center, New Orleans, LA; University Medical Center Burn Center, Louisiana State University Health Sciences Center, Department of Surgery, New Orleans, LA; UCI Health Regional Burn Center, New Orleans, LA; University Medical Center Burn Center, Louisiana State University Health Sciences Center, New Orleans, LA; University Medical Center Burn Center, Louisiana State University Health Sciences Center, New Orleans, LA; Louisiana State University Health Sciences Center, New Orleans, LA; Louisiana State University Health Sciences Center, New Orleans, LA; Louisiana State University Health Sciences Center, New Orleans, LA

## Abstract

**Introduction:**

Burn injuries induce profound metabolic disturbances, including hypermetabolism and altered lipid balance, which strongly influence recovery and clinical outcomes. Despite their importance, the mechanisms underlying fat metabolism after burn trauma remain poorly understood. Metabolomics, an analytical approach for profiling small molecules, provides a powerful tool to investigate these shifts. By characterizing lipid-related metabolic pathways, it may be possible to identify biomarkers and therapeutic targets that improve wound healing, modulate inflammation, and optimize nutritional strategies. This study aimed to investigate the lipidomic profiles of burn patients in response to injury.

**Methods:**

Fat was excised from adult patients with greater than 20% total body surface area (TBSA) during their index case. Control fat was obtained from patients undergoing elective breast reductions or abdominal wall liposuctions. Analysis employed an Agilent 8890 GC coupled with a 7250A Q-TOF MS, using a DB-5 ms column with helium and nitrogen as carrier and collision gases. Oven conditions ramped from 60°C to 325°C over 37.5 minutes. Electron ionization at 70 eV provided a mass range up to 3000 m/z. Data processing used MS-DIAL with FiehnLib for compound identification, and retention index normalization was performed with FAMEs.

**Results:**

Metabolomic profiling was conducted on tissue from 15 burn patients and 9 controls. Comparison of burn versus control groups revealed significant metabolic alterations. In total, 132 known compounds were identified. Burn patients demonstrated elevated levels of 5,6-Dihydrouracil, Beta-Alanine, Glutamate, Glycolic acid, Heptylaldehyde, Lactic acid, L-Methionine, Methanolphosphate, Proline, Succinic acid, Oxalic acid, and Urea. Statistical analysis confirmed these differences, suggesting distinct metabolic adaptations to thermal injury (*p*=.001–0.05).

**Conclusions:**

Burn trauma is associated with increased abundance of amino acids, organic acids, and aldehydes, reflecting complex systemic metabolic disruption. These metabolites may serve as candidate biomarkers to improve diagnostic and prognostic precision in burn care and could inform therapeutic strategies targeting metabolism, nutrition, and inflammation. Larger studies are warranted to validate these findings and further elucidate the biological mechanisms driving burn-associated metabolic shifts.

**Applicability of Research to Practice:**

This research highlights how burn injuries disrupt key metabolic pathways, identifying specific metabolites that may serve as biomarkers. Clinically, these findings can improve diagnostic accuracy, guide nutritional and therapeutic strategies, and enable earlier interventions to reduce complications. By linking metabolic shifts to patient outcomes, this work supports more precise and personalized burn care.

**Funding for the study:**

N/A.

